# Risk of appendicitis after SARS-CoV-2 infection and SARS-CoV-2 vaccination

**DOI:** 10.1093/bjs/znac127

**Published:** 2022-05-07

**Authors:** Rickard Ljung, Nicklas Pihlström, Anders Sundström

**Affiliations:** Division of Use and Information, Swedish Medical Products Agency, Uppsala, Sweden; Division of Use and Information, Swedish Medical Products Agency, Uppsala, Sweden; Division of Use and Information, Swedish Medical Products Agency, Uppsala, Sweden

*Dear Editor*

Appendicitis has been reported after SARS-CoV-2 infection and as a potential adverse reaction after SARS-CoV-2 vaccination^1–3^. The risk of appendicitis was evaluated after SARS-CoV-2 infection and SARS-CoV-2 vaccination compared with the risk in unvaccinated people by conducting a population-based cohort study in Sweden.

Some 9.5 million people aged 12 years and older were included, consisting of all residents on 1 January 2017, who were alive and still residing in the country on 27 December 2020^4^. A total of 72 722 people with any record of appendicitis from 1 January 2017 to 26 December 2020 were excluded. The risk of appendicitis in 21-day risk periods after administration of the first, second, and third dose with BNT162b2, mRNA-1273, and AZD1222 was evaluated. In a complementary analysis, incident appendicitis within 21 days after SARS-CoV-2 infection, from 1 August 2020 to the end of study on 31 January 2022, was studied.

The primary outcome was the date of first record of appendicitis (ICD-10 discharge diagnosis K35 in inpatient care), and the secondary outcome was appendicitis and related disorders (ICD-10 codes K35–K37) in either inpatient or outpatient care^5^. In a sensitivity analysis, a stricter definition was used, including both a record of appendicitis and a procedure code for appendicectomy.

The analysis was adjusted for sex, age, history of cystic fibrosis, diabetes, inflammatory bowel disease, antibiotic prescriptions, and SARS-CoV-2 infection. Poisson regression was used to estimate incidence rate ratios (IRRs) with 95 per cent confidence intervals, comparing rates in a 21-day interval after vaccination with rates in unvaccinated periods.

There were 126 episodes of appendicitis within 21 days among 999 289 individuals with verified SARS-CoV-2 infection, corresponding to 12.6 episodes per 100 000 individuals. Among 6 313 572 individuals vaccinated against SARS-CoV-2 with a first dose of mRNA vaccine, there were 410 episodes of appendicitis within 21 days of vaccination (6.5 per 100 000), 421 among 6 197 026 individuals (6.8 per 100 000) after second dose, and 142 among 3 782 468 individuals (3.8 per 100 000) after third dose (*Table 1*).

**Table 1:** Risk of appendicitis following SARS-CoV-2 vaccination and SARS-CoV-2 infection among 9.5 million persons aged 12 years and older

	Appendicitis using narrow definition (ICD-10 code K35)*		Appendicitis using broad definition (ICD–10 code K35-37)*	
	Event rate	Incidence rate ratio	No. of events	Incidence rate ratio
**First dose BNT162b2**				
Overall	348 of 5 427 708	0.97 (0.87, 1.08)	395	0.95 (0.86, 1.06)
Age 12–24 years	90 of 834 304	1.04 (0.85, 1.29)	109	1.09 (0.90, 1.32)
Age 25–44 years	95 of 1 518 176	0.79 (0.64, 0.97)	106	0.75 (0.62, 0.92)
Females	175 of 2 730 309	1.04 (0.89, 1.21)	194	0.98 (0.85, 1.14)
Males	173 of 2 697 399	0.91 (0.78, 1.06)	201	0.92 (0.80, 1.07)
**Second dose BNT162b2**				
Overall	350 of 5363897	1.00 (0.90, 1.12)	396	0.98 (0.88, 1.09)
Age 12–24 years	80 of 777921	1.01 (0.81, 1.27)	88	0.96 (0.78, 1.16)
Age 25–44 years	137 of 1503904	1.16 (0.97, 1.38)	150	1.09 (0.92, 1.28)
Females	177 of 2727873	1.06 (0.91, 1.24)	198	1.02 (0.,88, 1.18)
Males	173 of 2 636 024	0.95 (0.81, 1.10)	198	0.95 (0.82, 1.09)
**First dose mRNA-1273**				
Overall	62 of 885 864	0.99 (0.77, 1.27)	74	1.02 (0.81, 1.29)
Age 12–24 years	16 of 161 624	0.93 (0.57, 1.53)	21	1.06 (0.69, 1.62)
Age 25–44 years	29 of 310 975	1.17 (0.81, 1.70)	34	1.18 (0.84, 1.65)
Females	27 of 431 417	0.96 (0.66, 1.41)	33	1.00 (0.71, 1.42)
Males	35 of 454 447	1.02 (0.73, 1.42)	41	1.04 (0.76, 1.42)
**Second dose mRNA-1273**				
Overall	71 of 833 129	1.25 (0.99, 1.58)	83	1.26 (1.02, 1.57)
Age 12–24 years	22 of 124 517	1.67 (1.10, 2.65)	24	1.57 (1.05, 2.35)
Age 25–44 years	26 of 293 786	1.12 (0.76, 1.66)	33	1.22 (0.87, 1.72)
Females	35 of 413 211	1.33 (0.96, 1.86)	43	1.40 (1.04, 1.89)
Males	36 of 419 918	1.18 (0.85, 1.63)	40	1.14 (0.83, 1.56)
**First dose any mRNA vaccine**				
Overall	410 of 6313572	0.97 (0.88, 1.08)	469	0.96 (0.88, 1.06)
**Second dose any mRNA vaccine**				
Overall	421 of 619 7026	1.04 (0.94, 1.14)	479	1.02 (0.93, 1.12)
**Third dose any mRNA vaccine**				
Overall	142 of 3 782 468	0.89 (0.75, 1.05)	168	0.91 (0.78, 1.07)
**First dose AZD1222**				
Overall	37 of 712 353	1.05 (0.76, 1.46)	44	1.09 (0.81, 1.47)
**Second dose AZD1222**				
Overall	28 of 574 178	1.08 (0.74, 1.58)	33	1.11 (0.79, 1.57)
**SARS-CoV-2 infection**				
Overall	126 of 999 289	1.68 (1.41, 2.00)	151	1.73 (1.47.2.03)
Age 12–24 years	38 of 233 824	1.59 (1.16, 2.19)	47	1.66 (1.25, 2.22)
Age 25–44 years	62 of 388 630	1.99 (1.55, 2.56)	71	1.95 (1.54, 2.47)
Females	68 of 497 139	1.63 (1.26, 2.12)	84	1.84 (1.49, 2.29)
Males	58 of 502 150	1.72 (1.35, 2.18)	67	1.60 (1.26, 2.04)

Values in parentheses are 95 per cent confidence intervals. Results are presented overall for the entire study population 12 years and older by sex, and in two age subgroups (age 45 years and older not presented in subgroups). *Appendicitis (ICD code K35 in inpatient care), secondary outcome as appendicitis and related disorders (ICD code K35–K37) in either inpatient or outpatient care.

Adjusted IRRs in the 21-day risk period after a positive SARS-CoV-2 infection was 1.68 (95 per cent c.i. 1.41 to 2.00) compared with uninfected periods. Excluding positive SARS-CoV-2 infection on same day as admission of appendicitis yielded a lower IRR of 1.24 (1.01 to 1.52).

Adjusted IRRs were 0.97 (0.87 to 1.08) after a first dose of BNT162b2, 1.00 (0.90 to 1.12) after a second dose, and 0.89 (0.73 to 1.08) after a third dose. Adjusted IRRs were 0.99 (0.77 to 1.27) after a first dose of mRNA-1273, 1.25 (0.99 to1.58) after a second dose, and 0.87 (0.63 to 1.21) after a third dose. Using the secondary outcome definition and other sensitivity analyses yielded IRRs similar to those of the primary analyses. Subgroup analyses restricted to ages 12–24 and 25–44 years are presented separately.

In this nationwide study comprising 9.5 million individuals, there was an increased risk of appendicitis after SARS-CoV-2 infection, but no overall association between SARS-CoV-2 vaccination and appendicitis. The isolated finding of a slightly increased relative risk of appendicitis after vaccination with a second dose of mRNA-1273 should be interpreted with caution owing to the weak strength of the association and small number of events. Trials of the BNT162b2 vaccine have shown a slightly higher rate of appendicitis among vaccinated compared with placebo groups. Surveillance data from the USA found no association between SARS-CoV-2 vaccination and appendicitis, whereas data from Israel indicated an increased risk following vaccination with BNT162b2^2,3^. The magnitude of the observed increased risk after SARS-CoV-2 infection must be interpreted with caution as the association can be affected by the testing strategy. If only patients with severe COVID-19 are tested, the association with other events, such as appendicitis, will be strengthened owing to selection bias.

*Disclosure*. Dr Ljung reported receiving grants from Sanofi Aventis paid to his institution outside the submitted work; and receiving personal fees from Pfizer outside the submitted work. Mr Pihlström reported no conflict of interest. Dr Sundström reported participating in research funded by governmental agencies, universities, Astellas Pharma, Janssen Biotech, AstraZeneca, Pfizer, Roche, (then) Abbott Laboratories, (then) Schering-Plough, UCB Nordic, and Sobi, with all funds paid to Karolinska Institutet, outside the submitted work.
